# UFV-P2 as a member of the Luz24likevirus genus: a new overview on comparative functional genome analyses of the LUZ24-like phages

**DOI:** 10.1186/1471-2164-15-7

**Published:** 2014-01-03

**Authors:** Monique R Eller, Pedro M P Vidigal, Rafael L Salgado, Maura P Alves, Roberto S Dias, Cynthia C da Silva, Antônio F de Carvalho, Andrew Kropinski, Sérgio O De Paula

**Affiliations:** 1 Department of Food Technology, Federal University of Viçosa, Av. PH Rolfs, s/n, Campus da UFV, Viçosa, Minas Gerais 36570-000, Brazil; 2 Bioinformatics Laboratory, Institute of Applied Biotechnology to Agriculture (BIOAGRO), Federal University of Viçosa, Av. PH Rolfs, s/n, Campus da UFV, Viçosa, Minas Gerais 36570-000, Brazil; 3 Department of Biochemistry and Molecular Biology, Federal University of Viçosa, Av. PH Rolfs, s/n, Campus da UFV, Viçosa, Minas Gerais 36570-000, Brazil; 4 Laboratory of Molecular Immunovirology, Federal University of Viçosa, Av. PH Rolfs, s/n, Campus da UFV, Viçosa, Minas Gerais 36570-000, Brazil; 5 Department of General Biology, Federal University of Viçosa, Av. PH Rolfs, s/n, Campus da UFV, Viçosa, Minas Gerais 36570-000, Brazil; 6 Department of Microbiology, Federal University of Viçosa, Av. PH Rolfs, s/n, Campus da UFV, Viçosa, Minas Gerais 36570-000, Brazil; 7 Public Health Agency of Canada, Laboratory for Foodborne Zoonoses, Guelph, Ontario N1G 3 W4, Canada; 8 Department of Molecular and Cellular Biology, University of Guelph, Guelph, Ontario N1G 2 W1, Canada

## Abstract

**Background:**

Phages infecting spoilage microorganisms have been considered as alternative biocontrol agents, and the study of their genomes is essential to their safe use in foods. UFV-P2 is a new *Pseudomonas fluorescens*-specific phage that has been tested for its ability to inhibit milk proteolysis.

**Results:**

The genome of the phage UFV-P2 is composed of bidirectional modules and presented 75 functionally predict ORFs, forming clusters of early and late transcription. Further genomic comparisons of *Pseudomonas*-specific phages showed that these viruses could be classified according to conserved segments that appear be free from genome rearrangements, called locally collinear blocks (LCBs). In addition, the genome organization of the phage UFV-P2 was shown to be similar to that of phages PaP3 and LUZ24 which have recently been classified as a *Luz24likevirus*.

**Conclusions:**

We have presented the functional annotation of UFV-P2, a new *Pseudomonas fluorescens* phage. Based on structural genomic comparison and phylogenetic clustering, we suggest the classification of UFV-P2 in the *Luz24likevirus* genus, and present a set of shared locally collinear blocks as the genomic signature for this genus.

## Background

According to the International Committee of Virus Taxonomy (ICTV) classification scheme based on morphology, biological characteristics and genome organization (http://www.ictvonline.org/virusTaxonomy.asp), the bacteriophage family *Podoviridae* contains two subfamilies and 11 genera, and the *Luz24likevirus* genus comprises the *Pseudomonas*-infecting bacteriophages PaP3 [1] and LUZ24 [2]. Beyond PaP3 and LUZ24, the phages tf [3], MR299-2 [4], PaP4(KC294142), vB_PaeP_p2-10_Or1 (HF543949) and vB_PaeP_C1-14_Or (HE983844) have similar genomic compositions and should be classified to this genus.

*Pseudomonas fluorescens* bacteriophage UFV-P2 [5], is a virus with a high ability to reduce casein proteolysis in milk. Milk proteolysis is caused by thermo-resistant enzymes produced by psychrotrophs and is responsible for serious losses in the dairy industry due to negative effects on the quality and reduced shelf life of dairy products. In this environment, *Pseudomonas* spp. are prevalent contaminants [6-8], mainly *P. fluorescens*[9,10]. The use of phages in biocontrol has been suggested as an alternative to the use of chemicals. For example, *P. fluorescens*-specific phages had been studied to control *Pseudomonas* population and as sanitation agents to efficiently remove bacterial biofilms on stainless steel surfaces similar to those used in food industries, where these contaminants are common [11-13]. However, they must be used with caution. In addition to proteolysis reduction and biofilm inhibition studies and, their host range determination, it is necessary to understand phages’ genome and proteome to make possible their use as biocontrol agents.

To expand our understanding about the *P. fluorescens*-specific phage UFV-P2, we present in detail the analysis of its structural genome and its comparisons to other phage genomes.

## Methods

### Sampling

The phage UFV-P2 was isolated from wastewater of a dairy industry in Minas Gerais, Brazil, and propagated at 30°C in LB medium in a strain of *P. fluorescens* 07A, courtesy of Laboratory of Food Microbiol, located at the Federal University of Viçosa, Brazil.

### Genome extraction and composition

Phages were propagated in LB medium containing the bacteria in exponential phase. After incubation at 30°C for 8 h, particle assemble was induced with mitomicin and the virions were recovered by centrifugation and filtration. Phage suspensions were incubated with 75 μg/mL of proteinase K in the presence of 0.01% SDS at 56°C for 90 min. Proteins were removed by extraction with phenol, phenol:chloroform (1:1), followed by chloroform. Genetic material was concentrated with an equal volume of isopropanol and resuspended in 30 μL of distilled water. For analysis of viral genome composition, 5 μL of the genomic extracts were submitted to digestion assays with enzymes DNase I (50 μg/mL) or RNaseA (100 μg/mL) for 60 min at 37°C, followed by 1% agarose gel electrophoresis and visualization by staining with GelRed (Biotium, USA).

### Genomic DNA sequencing and assembly

UFV-P2 genome was sequenced using an Illumina Genome Analyzer II by CD Genomics (New York, USA) and was assembled and analyzed using CLC Genomics Workbench version 5.1 (CLC bio, Cambridge, MA, USA). The sequence reads were assembled into contigs using stringent parameters, in which 90% of each read had to cover the other read with 90% identity. The data are available in GenBank database under accession number JX863101.

### Bioinformatics analysis

The genome of phage UFV-P2 was oriented to be collinear with that of the type species, *Pseudomonas* phage LUZ24, and manually annotated using Kodon (Applied Maths, Austin, TX, USA.) [14]. The GenBank flat file (gbk) file was exported from Kodon and converted to FASTA-formatted protein sequences using gbk2faa (http://lfz.corefacility.ca/gbk2faa/). The latter were screened for viral homologs using the BLASTP feature of Geneious R6.1 (Biomatters Ltd., Auckland, New Zealand); and, for protein motifs, using TMHMM [15], Phobius [16] and Batch Web CD-Search Tool [17] at http://www.ncbi.nlm.nih.gov/Structure/bwrpsb/bwrpsb.cgi.

Putative promoters were identified using the Kodon sequence similarity search feature employing TTGACA(N15-18)TATAAT and allowing for a 2 bp mismatch. Rho-independent terminators were tentatively identified using ARNold [18,19] at http://rna.igmors.u-psud.fr/toolbox/arnold/index.php.

For comparative purposes at the genomic level EMBOSS Stretcher [20] and progressive Mauve [21] were employed; while at the proteomic level we used CoreGenes [22,23]. Seventeen genomic reference sequences of phages were downloaded from GenBank (Table 1) and compared to UFV-P2 genome.

**Table 1:** Pairwise comparisons of phage UFV-P2 and others phage genomes

				**Phage UFV-P2**	
**Phage**	**GenBank accession**	**GC content (%)**	**Genome density (genes/kbp)**	**Identities**	**%**
**UFV-P2**	JX863101	51.5	1.65	-	-
**vB_PaeP_p2-10_Or1**	HF543949	52.0	1.32	27,253	57.46
**vB_PaeP_C1-14_Or**	HE983844	52.0	1.41	27,672	57.31
**LUZ24**	NC_010325	52.2	1.49	27,510	56.80
**PaP4**	KC294142	52.5	1.59	27,015	56.73
**PaP3**	NC_004466	52.2	1.56	27,358	56.20
**MR299-2**	JN254801	52.0	1.52	27,192	56.19
**Tf**	NC_017971	53.2	1.51	23,750	49.55
**Phi-2**	NC_013638	58.9	1.00	22,672	46.73
**phiKMV**	NC_005045	62.3	1.13	22,392	46.43
**phiIBB-PF7A**	NC_015264	56.3	1.27	22,150	46.41
**Bf7**	NC_016764	58.4	1.15	21,825	46.16
**PaP2**	NC_005884	45.4	1.32	22,523	46.11
**119X**	NC_007807	44.9	1.29	22,434	46.03
**T7 ( *Enterobacteria *)**	NC_001604	48.4	1.50	21,602	45.66
**gh-1**	NC_004665	57.4	1.12	21,218	45.35
**F116**	NC_006552	63.2	1.07	27,102	41.20
**LUZ7**	NC_013691	53.2	1.54	29,160	38.74

### Phylogenetic clustering

For clustering UFV-P2 phage in an evolutionary way, a phylogenetic hypothesis was inferred by Bayesian inference (BI) using MrBayes v3.2.2 [24]. The genomic sequences of phages were aligned using ClustalW [25], and a pairwise distance matrix was calculated MEGA version 5 [26] (Table 1). The alignment was manually inspected, and the sites with gaps were excluded. To expedite the construction of phylogenetic trees, a model of nucleotide substitution was estimated using the jModelTest 2 program [27]. The GTR + G substitution model was selected as the best DNA evolution model for genomic sequences, according to the Akaike Information Criterion (AIC) and Bayesian Information Criterion (BIC).

The BI phylogenetic tree was calculated using the Bayesian Markov Chain Monte Carlo (MCMC) method, in two runs with 5,000,000 generations. The convergence of the parameters was analyzed in TRACER v1.5.0 (http://beast.bio.ed.ac.uk/tracer), and the chains reached a stationary distribution after 50,000 generations. Then, a total of 1% of the generated trees was burned to produce the consensus tree. To root the phylogenetic tree, the Enterobacteria phage T7 (NC_001604) was selected as outgroup taxa.

## Results and Discussion

Transmission electron microscopy of the UFV-P2 virions (data not shown) showed that this virus has isometric capsids and very short tails, with morphological similarity to the *P. aeruginosa* phages Pap3 and MR299-2. Thus, UFV-P2 can be inserted in the *Podoviridae* family, order *Caudovirales.*

### Functional genomic organization

#### The viral genome was extracted and sequenced

The phage UFV-P2 has a linear 45,517 bp DNA genome with a GC content of 51.5%, and was sequenced with coverage of 30,655 fold. One of the interesting characteristics of members of the *Luz24likevirus* genus is the presence of localized single-stranded breaks associated with the consensus sequence TACTRTGMC [28]. Fourteen of these sequences were found in the top strand of the tf DNA, while the genome of UFV-P2 contains 15.

**Figure 1:**
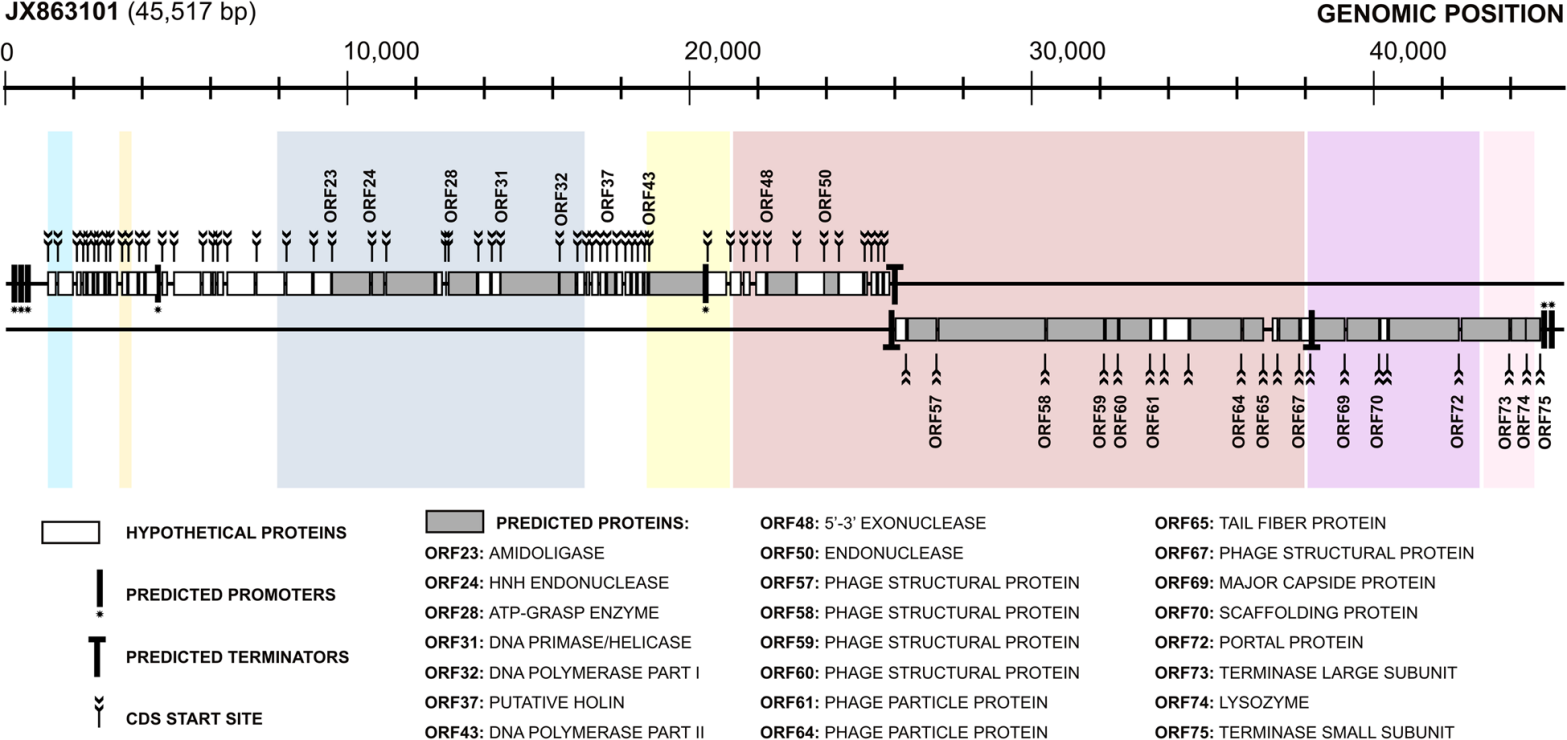
**The genomic organization of the phage UFV-P2.** The colored squares in the background correspond to the conserved locally collinear blocks (LCB) found for the UFV-P2 in relation to the other members of the *Luz24likevirus* genus (Figure 2). The detailed annotation is shown in Table 2.

At first, bioinformatics analyses had showed that the UFV-P2 genome has a bidirectional organization with 92 predicted open reading frames (ORFs) larger than 100 bp, but only 41 ORFs (44.75%) could be identified as coding sequences (CDS) by similarity searches against known proteins in the GenBank and UniProt databases [5]. However, we propose a new annotation of the genome of this virus based on different tools, which were able to functionally predict 75 ORFs also bidirectionally oriented and forming clusters of early and late transcription (Figure 1 and Table 2).

**Table 2:** Functional genomic annotation of phage UFV-P2

**Gene**	**Predicted protein**	**Genomic coordinates**	**Strand**	**Protein mass (Da)**	**Protein pI**	**AA residues**	**Homologs (a) & Motifs**	**E-value**
ORF01	Conserved hypothetical protein	1205..1483	+	10486	10.5	92	NP_955002 hypothetical protein PaP3p71 [Pseudomonas phage PaP3]	7.34E-09
ORF02	Conserved hypothetical protein	1501..1983	+	17752	9.4	160	YP_006382463 hypothetical protein tf_02 [Pseudomonas phage tf]; protein motifs: cl10333 PHA01782	3.22E-37
ORF03	Conserved hypothetical protein	2046..2216	+	6515	7.8	56	YP_007183264 hypothetical protein BN425_ORF_56 [Pseudomonas phage vB_PaeP_p2-10_Or1]	7.83E-02
ORF04	Hypothetical protein	2231..2368	+	5276	9.8	45	-	-
ORF05	Hypothetical protein	2365..2556	+	7171	9.2	63	-	-
ORF06	Hypothetical membrane protein	2553..2687	+	4708	9.5	44	protein motifs: one transmembrane domain discovered using TMHMM and Phobius	
ORF07	Conserved hypothetical protein	2677..2898	+	8374	9.8	73	YP_006382470 hypothetical protein tf_10 [Pseudomonas phage tf]	2.09E-04
ORF08	Conserved hypothetical protein	2898..3020	+	5178	9.3	40	AGC35239 hypothetical protein PaP4_008 [Pseudomonas phage PaP4]	1.19E-05
ORF09	Hypothetical protein	3021..3275	+	9120	4.1	84	-	-
ORF10	Hypothetical protein	3368..3568	+	7493	9.6	66	-	-
ORF11	Hypothetical protein	3555..3869	+	11403	5.0	104	-	-
ORF12	Hypothetical protein	3869..4063	+	7139	9.0	64	-	-
ORF13	Conserved hypothetical protein	4060..4458	+	14906	9.4	132	YP_006382473 hypothetical protein tf_13 [Pseudomonas phage tf]	9.55e-15
ORF14	Hypothetical membrane protein	4539..4736	+	7618	9.1	65	protein motifs: one to two transmembrane domains discovered using TMHMM and Phobius	
ORF15	Conserved hypothetical protein	4884..5714	+	29824	8.5	276	AGC35249 transposase fusion protein [Pseudomonas phage PaP4]; protein motifs: pfam01145 Band_7, & cl02525 Band_7	1.15E-113
ORF16	Conserved hypothetical protein	5727..6017	+	10708	9.1	96	YP_006659979 hypothetical protein tf_14 [Pseudomonas phage tf]	2.29E-11
ORF17	Conserved hypothetical protein	6021..6149	+	4783	11.0	42	NP_775206 hypothetical protein PaP3p51 [Pseudomonas phage PaP3]	1.32E-03
ORF18	Hypothetical protein	6159..6374	+	7803	5.0	71	-	-
ORF19	Conserved hypothetical protein	6441..7289	+	31291	9.2	282	YP_006382477 hypothetical protein tf_17 [Pseudomonas phage tf]	1.75E-03
ORF20	Conserved hypothetical protein	7296..8159	+	32906	4.9	287	YP_006382478 hypothetical protein tf_18 [Pseudomonas phage tf]	2.06E-07
ORF21	Conserved hypothetical protein	8171..8968	+	29423	5.0	265	YP_006382479 hypothetical protein tf_19 [Pseudomonas phage tf]; protein motifs: PF14395.1 COOH-NH2_lig	8.75E-112
ORF22	Conserved hypothetical protein	8965..9525	+	21203	7.5	186	NP_775210 hypothetical protein PaP3p47 [Pseudomonas phage PaP3]	2.53E-40
ORF23	Amidoligase	9501..10658	+	43407	5.5	385	YP_001671891 hypothetical protein [Pseudomonas phage LUZ24]; protein motifs: PF12224.3 Amidoligase_2	4.46E-96
ORF24	HNH endonuclease	10667..11071	+	15340	9.6	134	YP_002003475 gp2.8 [Enterobacteria phage BA14]; protein motifs: PF13392.1 HNH_3	2.71E-20
ORF25	Glutamine amidotransferase	11084..12544	+	54036	5.9	486	YP_006382482 glutamine amidotransferase [Pseudomonas phage tf]; protein motifs: PF13522.1 GATase_6, & cd00352 Gn_AT_II	2.72E-151
ORF26	Conserved hypothetical protein	12547..12765	+	8166	4.8	72	NP_775213 hypothetical protein PaP3p44 [Pseudomonas phage PaP3]	2.25E-20
ORF27	Hypothetical protein	12805..12909	+	3505	5.3	34	-	-
ORF28	ATP-grasp enzyme	12906..13781	+	31797	7.7	291	YP_006382486 hypothetical protein tf_24 [Pseudomonas phage tf]	2.60E-96
ORF29	Conserved hypothetical protein	13774..14169	+	15076	5.5	131	AGC35259 hypothetical protein PaP4_028 [Pseudomonas phage PaP4]; proteins motifs: PF06094.7 AIG2, & cd06661 GGCT_like	5.55E-27
ORF30	Conserved hypothetical protein	14169..14465	+	11295	5.4	98	YP_006382488 hypothetical protein tf_026 [Pseudomonas phage tf]	6.03E-23
ORF31	DNA primase/helicase	14425..16176	+	65673	5.9	583	YP_001671897 primase/helicase [Pseudomonas phage LUZ24]; protein motifs: PF13155.1 Toprim_2, & PF03796.10 DnaB_C	0
ORF32	DNA polymerase part I	16151..16666	+	20133	4.5	171	YP_006382490 3′-5′ exonuclease [Pseudomonas phage tf] & YP_001671898 DNA polymerase part I [Pseudomonas phage LUZ24]	2.82E-75; 3.72e-68
ORF33	Conserved hypothetical membrane protein	16669..16920	+	9085	10.0	83	YP_006382491 hypothetical protein tf_29 [Pseudomonas phage tf]; protein motifs: one transmembrane domain discovered using TMHMM and Phobius	1.99E-06
ORF34	Hypothetical protein	16931..17068	+	5096	3.4	45	-	-
ORF35	Hypothetical protein	17095..17325	+	8505	9.0	76	-	-
ORF36	Conserved hypothetical protein	17336..17539	+	7596	5.8	67	YP_001671900 hypothetical protein [Pseudomonas phage LUZ24]	2.23E-02
ORF37	Putative holin	17536..17817	+	10475	9.8	93	YP_006382493 hypothetical protein tf_32 [Pseudomonas phage tf] & YP_001671904 putative holin [Pseudomonas phage LUZ24]; protein motifs: three transmembrane domains discovered using TMHMM and Phobius	5.03e-35; 1.92e-18
ORF38	Hypothetical membrane protein	17814..18032	+	8079	9.9	72	protein motifs: one or two transmembrane domains discovered using TMHMM and Phobius	
ORF39	Conserved hypothetical protein	18069..18263	+	6728	3.5	64	YP_006382494 hypothetical protein tf_34 [Pseudomonas phage tf]	6.48E-04
ORF40	Conserved hypothetical protein	18263..18433	+	5628	8.2	56	YP_007112538 hypothetical protein MAR_61 [Vibrio phage vB_VpaM_MAR]	9.20E-05
ORF41	Conserved hypothetical protein	18433..18627	+	7272	10.9	64	YP_006382495 hypothetical protein tf_35 [Pseudomonas phage tf]	1.52E-03
ORF42	Hypothetical protein	18630..18764	+	4887	6.0	44	-	-
ORF43	DNA polymerase part II	18765..20405	+	60865	9.6	546	YP_007183240 DNA polymerase1 [Pseudomonas phage vB_PaeP_p2-10_Or1] & YP_001671907 DNA polymerase part II [Pseudomonas phage LUZ24]; protein motifs: PF00476.15 DNA_pol_A	0; 0
ORF44	DNA binding protein	20472..21068	+	21853	4.8	198	YP_006382500 DNA binding protein [Pseudomonas phage tf]	6.61E-70
ORF45	Conserved hypothetical protein	21139..21495	+	13200	6.3	118	AGC35272 hypothetical protein PaP4_041 [Pseudomonas phage PaP4]	4.02E-47
ORF46	Hypothetical protein	21525..21755	+	8335	4.7	76	-	-
ORF47	Hypothetical protein	21887..22222	+	12649	9.4	111	-	-
ORF48	5′-3′ exonuclease	22219..23103	+	33498	5.3	294	NP_775229 exonuclease [Pseudomonas phage PaP3]; protein motifs: PF01367.15 5_3_exonuc, & cd09898 H3TH_53EXO	1.47E-149
ORF49	Conserved hypothetical protein	23078..24073	+	37154	4.7	331	YP_006659984 conserved hypothetical protein [Pseudomonas phage tf]	1.15E-39
ORF50	Endonuclease	23874..24341	+	17655	5.4	155	YP_006382505 endonuclease [Pseudomonas phage tf]	1.11E-41
ORF51	Conserved hypothetical protein	24307..25062	+	28981	6.2	251	YP_001671917 hypothetical protein [Pseudomonas phage LUZ24]	2.15E-129
ORF52	Hypothetical protein	25059..25184	+	4844	10.5	41	-	-
ORF53	Conserved hypothetical protein	25255..25458	+	7717	6.5	67	YP_006659986 hypothetical protein tf_48 [Pseudomonas phage tf]	7.14E-14
ORF54	Hypothetical protein	25455..25631	+	6814	4.6	58	-	-
ORF55	Conserved hypothetical protein	25628..25834	+	8109	4.4	68	YP_001671920 hypothetical protein [Pseudomonas phage LUZ24]	1.57E-12
ORF56	Conserved hypothetical protein	25963..26319	-	13124	5.9	118	AGC35282 hypothetical protein PaP4_051 [Pseudomonas phage PaP4]	2.11E-45
ORF57	Phage structural protein	26321..27208	-	32029	5.5	295	AFD10698 hypothetical protein I7C_020 [Pseudomonas phage MR299-2]	3.43E-152
ORF58	Phage structural protein	27219..30383	-	111420	5.3	1054	YP_001671923 phage particle protein [Pseudomonas phage LUZ24]	0
ORF59	Phage structural protein	30389..32101	-	60235	5.5	570	YP_001671924 phage particle protein [Pseudomonas phage LUZ24]	1.35E-86
ORF60	Phage structural protein	32103..32507	-	13485	6.5	134	YP_001671925 phage particle protein [Pseudomonas phage LUZ24]	9.26E-11
ORF61	Phage particle protein	32504..33448	-	32240	4.8	314	AFD10694 hypothetical protein I7C_016 [Pseudomonas phage MR299-2]	3.23E-112
ORF62	Conserved hypothetical protein	33429..33863	-	16522	4.5	144	AGC35288 hypothetical protein PaP4_057 [Pseudomonas phage PaP4]	2.95E-57
ORF63	Conserved hypothetical protein	33860..34570	-	25585	4.8	236	YP_001671928 hypothetical protein [Pseudomonas phage LUZ24]	3.73E-56
ORF64	Phage particle protein	34570..36111	-	57542	5.1	513	YP_006382515 phage particle protein [Pseudomonas phage tf] & YP_001671929 hypothetical protein [Pseudomonas phage LUZ24]	0; 0
ORF65	Tail fiber protein	36119..36754	-	21879	6.7	211	NP_775246 hypothetical protein PaP3p12 [Pseudomonas phage PaP3]	1.68E-62
ORF66	Conserved hypothetical protein	36976..37170	-	6838	6.6	64	YP_001671932 hypothetical protein LUZ24 [Pseudomonas phage LUZ24]	3.02E-28
ORF67	phage structural protein	37175..37804	-	23887	5.0	209	AFD10687 putative constituent protein [Pseudomonas phage MR299-2]	2.54E-82
ORF68	Conserved hypothetical protein	37808..38128	-	11847	5.8	106	YP_007183215 hypothetical protein BN425_ORF_07 [Pseudomonas phage vB_PaeP_p2-1 0_Or1]	1.26E-50
ORF69	Major capsid protein	38180..39133	-	34740	6.0	317	NP_775251 major head protein [Pseudomonas phage PaP3] & YP_001671935 major head protein [Pseudomonas phage LUZ24]	0; 0
ORF70	Scaffolding protein	39152..40138	-	36190	4.3	328	YP_001671936 scaffolding protein [Pseudomonas phage LUZ24]	4.90E-101
ORF71	Conserved hypothetical protein	40128..40379	-	9559	5.2	83	AGC35298 hypothetical protein PaP4_067 [Pseudomonas phage PaP4] & YP_001671937 hypothetical protein [Pseudomonas phage LUZ24]	8.9E-30; 2.00e-29
ORF72	Portal protein	40379..42469	-	79405	5.0	696	YP_007183212 putative portal protein [Pseudomonas phage vB_PaeP_p2-10_Or1] YP_001671938 portal protein [Pseudomonas phage LUZ24]	0; 0
ORF73	Terminase, large subunit	42496..43941	-	54330	6.0	481	YP_001671939 terminase large subunit [Pseudomonas phage LUZ24]; protein motifs: PF03237.10 Terminase_6	0
ORF74	Lysozyme	43945..44451	-	18961	8.6	168	YP_006382529 lysozyme [Pseudomonas phage tf]; protein motifs: PF00959.14 Phage_lysozyme	3.27E-56
ORF75	Terminase, small subunit	44372..44845	-	17291	5.9	157	YP_007183209 hypothetical protein BN425_ORF_01 [Pseudomonas phage vB_PaeP_p2-1 0_Or1] & YP_006382530 terminase small subunit [Pseudomonas phage tf]	5.76E-66; 2.59e-64

The searches for consensus sequences of transcriptional promoters revealed the presence of seven promoters, five in the positive strand initiating the transcription of ORFs that encode early proteins, which is a common feature of viral genomes that need bacterial transcription factors to start their infection cycle. The two other promoters are located in late genes modules. These genes are usually transcribed by viral transcription factors.

Three rho-independent transcription terminators were predicted using ARNold, one in the positive and two in the negative strand (Figure 1). A bidirectional termination region was found in the region from 25,922 to 25,964. Interestingly, this pattern of termination is also found in the genomes of the phages PaP3 [1] and LUZ24 [2]. The last terminator sequence is located at the terminal end of the gene encoding the major head protein. The low number of sequences of rho-independent terminators compared to the number of predicted ORFs may be due to the existence of other types of terminators or the presence of transcriptional modules and the generation of polycistronic mRNAs, a very common feature of viral genomes.

The predicted UFV-P2 genes were functionally classified as its promoters, predicted order of transcription, and its annotated functions.

#### Nucleotide biosynthesis and DNA replication (positive-stranded ORFs)

Fifty-five genes (ORFs 01–55) involved in nucleotide biosynthesis and viral replication process were found in the UFV-P2 genome positive strand, named early genes (Figure 1). Among viral replication genes, ORF31 encodes a primase/helicase; ORF44, a DNA-binding protein; ORF48, a 5′-3′ exonuclease; ORF50, a putative endonuclease; and ORFs 32 and 43 encode the two exons of the viral DNA polymerase, between which there is an ORF encoding a putative holin with three transmembrane domains similar to those from the phages tf and LUZ24. Holins are small membrane proteins that accumulate in the membrane until, at a specific time that is “programmed” into the holin, the membrane suddenly becomes permeabilized to the fully folded endolysin [29]. In addition, the UFV-P2 genome contains two endonucleases encoded by ORF24 and ORF50. The first is a HNH endonuclease, a group I homing endonuclease, which may be related to the presence of introns in the UFV-P2 genome [30], like those between the two parts of DNA polymerase. Other enzymes predicted in the positive strand include ORFs 23, 25 and 28, which encode, respectively, an amidoligase, a glutamine amidotransferase and an ATP-grasp enzyme. The other 45 proteins of the early genes module are hypothetical proteins.

#### Virion assembly and host lysis (negative-stranded ORFs)

Twenty genes (ORFs 56–75) related to the composition and assembly of the viral particle, DNA packaging, and host lysis were found in the UFV-P2 genome negative strand, named late genes (Figure 1). Two transcriptional modules were found based on predicted terminators. The first is located in the regions comprising the ORFs 75–69, and the second module corresponding to the ORFs 75–56.

In the first module, ORF75 and ORF73 encode the small and large terminase subunits, respectively. The terminase is the motor component that assists the translocation of viral genomic DNA to the inner of the capsid during packaging via ATP hydrolysis. There is an ongoing discussion about the role of terminase structure in determining the points for cleavage of the viral DNA, which would influence the entire viral genome organization [31]. Recently, Shen and coworkers [32] functionally identified the two genes encoding PaP3 terminase subunits, located in ORFs 1 and 3, respectively, which have high sequence similarity with ORFs 75 and 73 of the UFV-P2 genome. The PaP3 genome have been annotated as opposing transcriptional gene clusters in relation to the UFV-P2 genome, what explains the difference observed for the numbering of similar ORFs. The same occurred for the earlier annotation of phage UFV-P2 [5], which is revised in this work to correspond to the annotation of phage LUZ24, which represents the genus.

ORF72 encodes the portal protein; ORF69 encodes the major head protein; and ORF70 encodes a scaffolding protein, which is a chaperone possibly related to viral particle assembly. In the second module, beyond the ORFs from the first, the ORFs 57–61, 64 and 67 encode particle/structural proteins; ORF65 encodes the tail fiber protein; and the other six ORFs encode hypothetical proteins.

ORF74 encodes a lysozyme that is used in the process of host cell breakage through the lysis of the peptidoglycan layer. The occurrence of a lysin, not associated with its cognate holin, is unusual but also found in other members of the *Luz24likevirus* genus.

### Structural genomic comparisons and evolutionary clustering

Pairwise genomic comparisons has been a useful approach for genotyping and classification of viruses like *Circoviridae*[33] and *Geminiviridae*[34]. The alignment of phages genomic sequences and pairwise comparisons revealed that vb_PaeP_p2-10_Or1, vb_PaeP_C1-14_Or, LUZ24, PaP4, PaP3, MR299-2 and tf are the phages most closely related to UFV-P2. Genomic sequences of these phages presented an identity to the UFV-P2 genome ranging from 49.5% to 57.5% (see Table 1).

**Figure 2:**
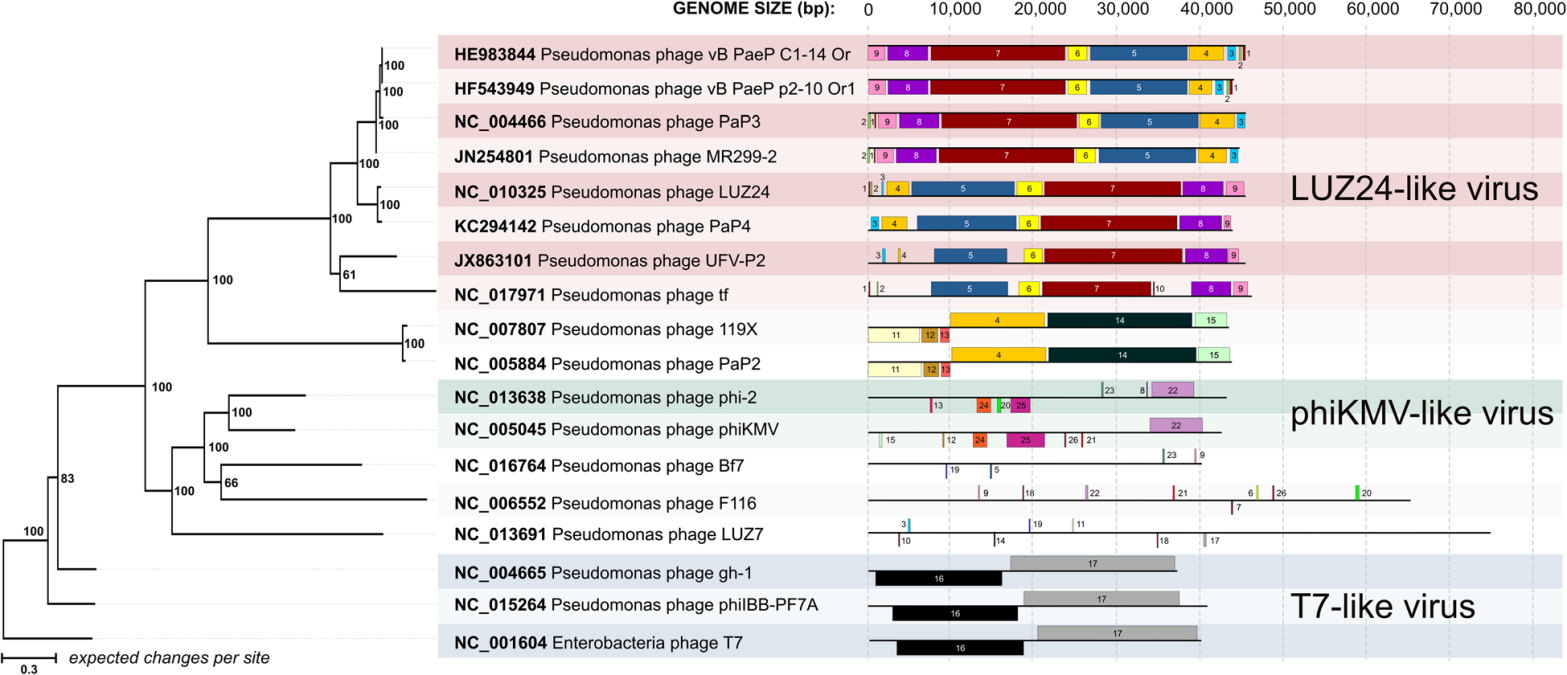
**Phylogenetic clustering and structural genomic comparisons among the UFV-P2 and other phages.** Phylogenetic tree of phage genomes (left) was calculated by Bayesian MCMC coalescent analysis. The posterior probability values (PP) (expressed as percentages) calculated using the best trees found by MrBayes are shown beside each node. The outgroup taxon is the Enterobacteria phage T7 (NC_001604). The colored squares in the schematic view of genomes (right) correspond to the conserved locally collinear blocks (LCBs) predicted by Mauve. The numbers and colors indicate the LCBs that are shared between the phages genomes.

**Figure 3:**
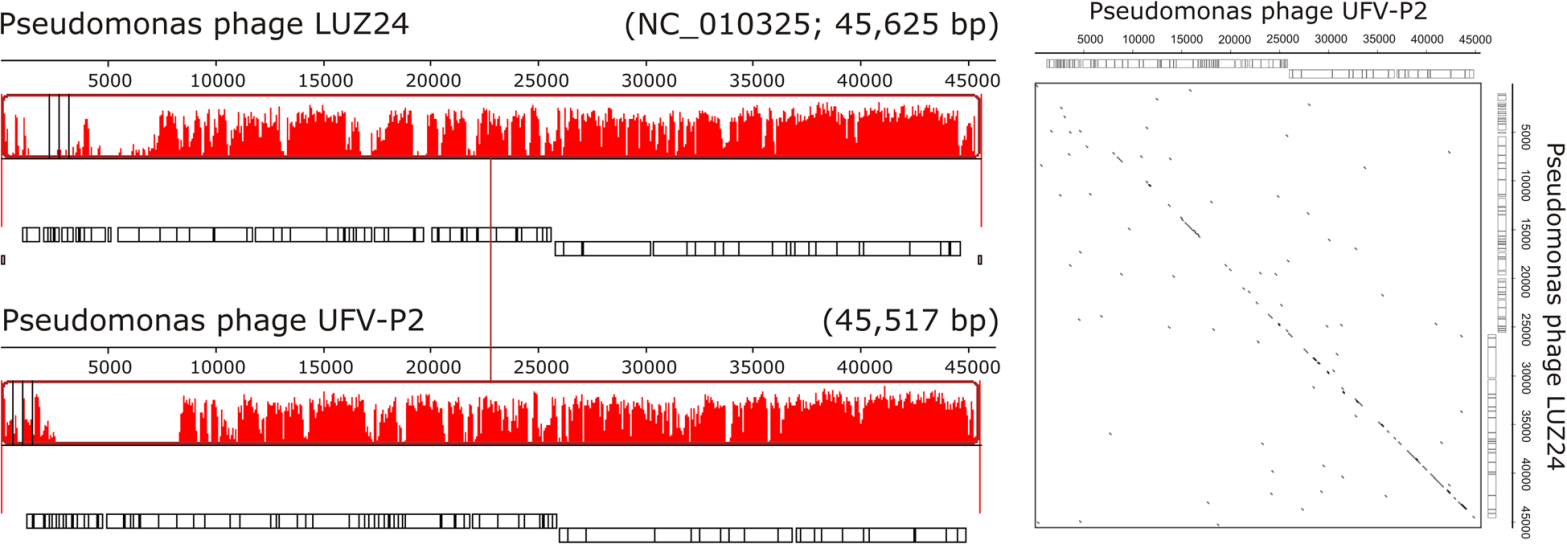
**Comparison of the genomes of the phages UFV-P2 and LUZ24.** The collinearity between genomes is represented by the conserved locally collinear block (left) and Dot plot alignment (right). Dot plot alignment was calculated using Nucleic Acid Dot Plots (http://www.vivo.colostate.edu/molkit/dnadot/index.html), considering a window size of 13 and a mismatch limit of 0.

The structural genomic comparisons in Mauve showed that these phages shared a set of conserved locally collinear blocks (LCB) (Figure 2 and Additional file 1: Figure S2). LCBs are conserved segments that appear be free from genome rearrangements, since the orthologous regions of genomes can be reordered or inverted by recombination processes [21]. In addition, a specific comparison between UFV-P2 and LUZ24 showed colinearity across their genomes (Figures 2 and 3).

Phages LUZ24, PaP4, and UFV-P2 present a conserved bidirectional genomic organization, which is showed by the shared LCBs (blocks 3–9) (Figure 2). Phage tf also presents this organization, but with some differences in the shared LCBs. On the other hand, phages MR299-2, PaP3, vb_PaeP_p2-10_Or1, and vb_PaeP_C1-14_Or present an inverted set of LCBs (blocks 9–3), representing an opposing arrangement of the gene modules. Proteins of these seven phages were the top hits with the UFV-P2 sequences (Table 2) and can collaborate with each other’s functional annotations. In addition to genomic comparisons, a search for direct terminal repeats (DTRs) indicated the presence of patterns at the ends of the UFV-P2 genome, as described for the phages LUZ24, tf, and vB_PaeP_C1-14_Or1. These repeats are responsible for the recognition and cleavage of the phage genome at the end of the repeat region during packaging. Interestingly, one of the unique features of this group of phages is that PaP3 possesses 20 bp 5′-protuding cohesive ends [1], while LUZ24 has 184 bp DTRs, yet there does not appear to be a significant difference in the amino acid sequence of their terminases.

As suggested by the structural genomic comparisons, phylogenetic tree of genomic sequences grouped the phages according the shared LCBs (Figure 2). Phages PaeP_p2-10_Or1, vb_PaeP_C1-14_Or, LUZ24, PaP4, PaP3, MR299-2, tf, and UFV-P2 were included in a distinct monophyletic clade in BI phylogenetic tree, which possibly represents the *Luz24likevirus* genus. The shared LCBs, blocks 3–9 (Figure 2), may be considered as a genomic signature for this genus. In UFV-P2 genome (Figure 1), as for the other phages, the genes for biosynthesis and DNA replication are included in blocks 5 and 6; genes for virion structure and assembly are in blocks 7 and 8; and genes for host lysis are block 9. In blocks 3 and 4 are included only hypothetical genes. Then, we propose the classification of the phage UFV-P2 in the *Luz24likevirus* genus. In fact, these analyzes showed that other viruses were also grouped in distinct monophyletic clades or according to specific shared locally collinear blocks (LCB), as those from the *T7likevirus* (blocks 16 and 17) and *Phikmvlikevirus* (blocks 22, 24, and 25) genera, beyond a possibly genus including the phages PaP2 and 199X (blocks 4 and 11–15).

## Conclusions

We have presented the functional annotation of UFV-P2, a new *Pseudomonas fluorescens* phage. Based on structural genomic comparison and phylogenetic clustering, we suggest the classification of UFV-P2 in the *Luz24likevirus* genus, and present a set of shared locally collinear blocks as the genomic signature for this genus.

## Competing interests

The authors declare that they have no competing interests.

## Authors’ contributions

MRE carried out the phage isolation, propagation, DNA extration, participated in the sequence alignment and drafted the manuscript. PMPV and RLS carried out the bioinformatic analysis and drafted the manuscript. MPA and RSD participated in the design of the study and initial processes of phage isolation, propagation, DNA extration and analysis. CCS and AFC were essential on the concept of the study, and participated in its design and coordination. AK participated in the bioinformatic analysis and concepts of phage classification. SOP is the responsible for the design and coordination of the project. All authors read and approved the final manuscript.

## Supplementary Material

Additional file 1: Figure S1 Transmission Electron Microscopy of the phage UFV-P2. Virions have isometric capsids of 40-50 nm and very short tails (arrows). Scale bars = 100 nm. Figure S2. Comparison of the genomes of phages classified in LUZ24likevirus genus. The collinearity among genomes is represented by the conserved locally collinear blocks (LCBs). In the main block (blue), the regions of similarity plot with high identity corresponds to the set of shared LCBs (see Figure 2). The connection line between blocks correspond to the central point of LCB of the reference genome (phage LUZ24 genome). Click here for file
