# Supplementary material for: UFV-P2 as a member of the Luz24likevirus genus: a new overview on comparative functional genome analyses of the LUZ24-like phages
Source: BMC Genomics. 2014 Jan 3;15:7. doi: 10.1186/1471-2164-15-7 (PMC3890496; doi:10.1186/1471-2164-15-7)
Supplement: Additional file 1: Figure S1 — Transmission Electron Microscopy of the phage UFV-P2. Virions have isometric capsids of 40-50 nm and very short tails (arrows). Scale bars = 100 nm. Figure S2. Comparison of the genomes of phages classified in LUZ24likevirus genus. The collinearity among genomes is represented by the conserved locally collinear blocks (LCBs). In the main block (blue), the regions of similarity plot with high identity corresponds to the set of shared LCBs (see Figure 2). The connection line between blocks correspond to the central point of LCB of the reference genome (phage LUZ24 genome). [file 1471-2164-15-7-S1.docx]

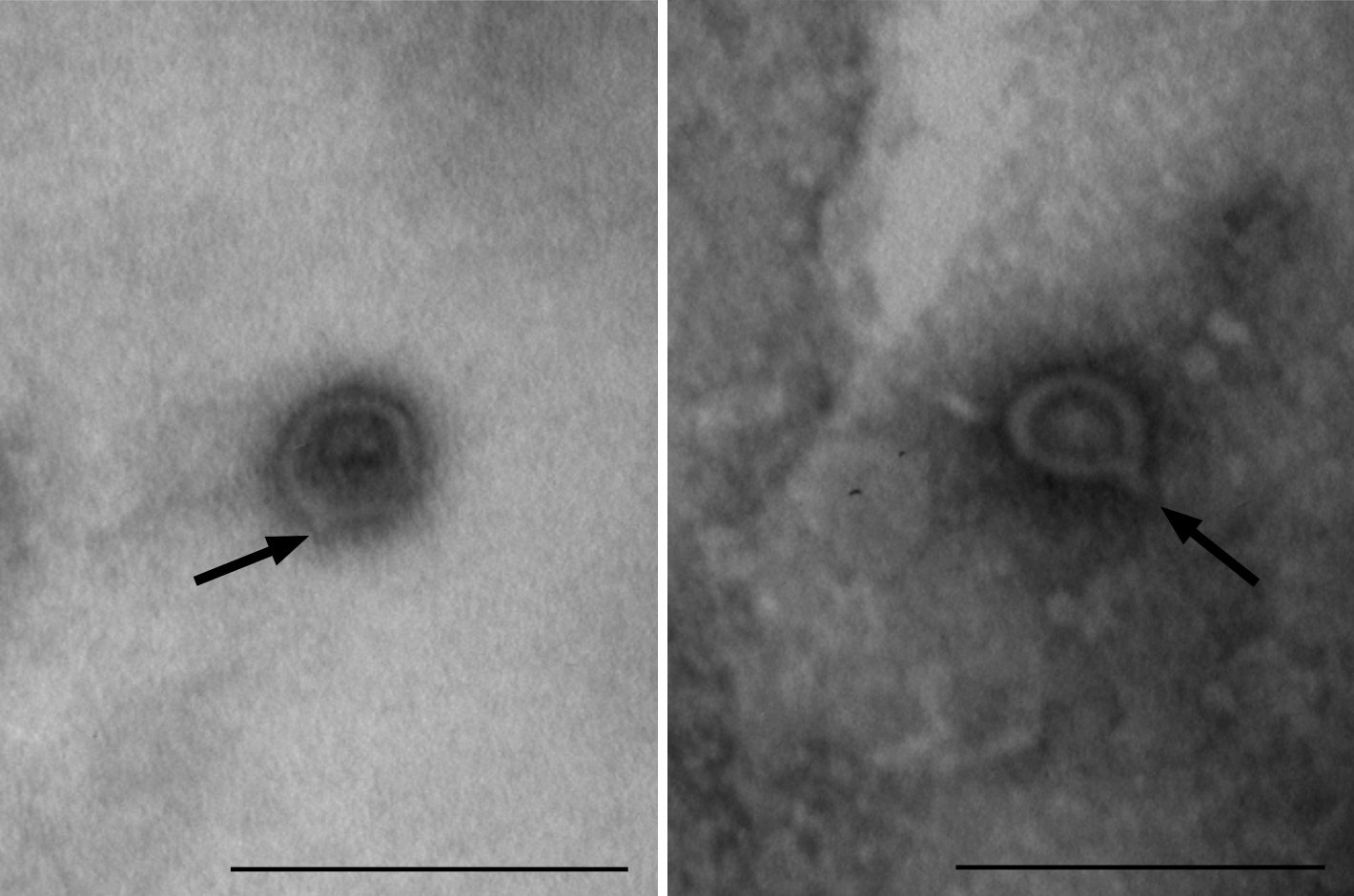


**Figure S1.** Transmission Electron Microscopy of the phage UFV-P2. Virions have isometric capsids of 40-50 nm and very short tails (arrows). Scale bars = 100 nm.


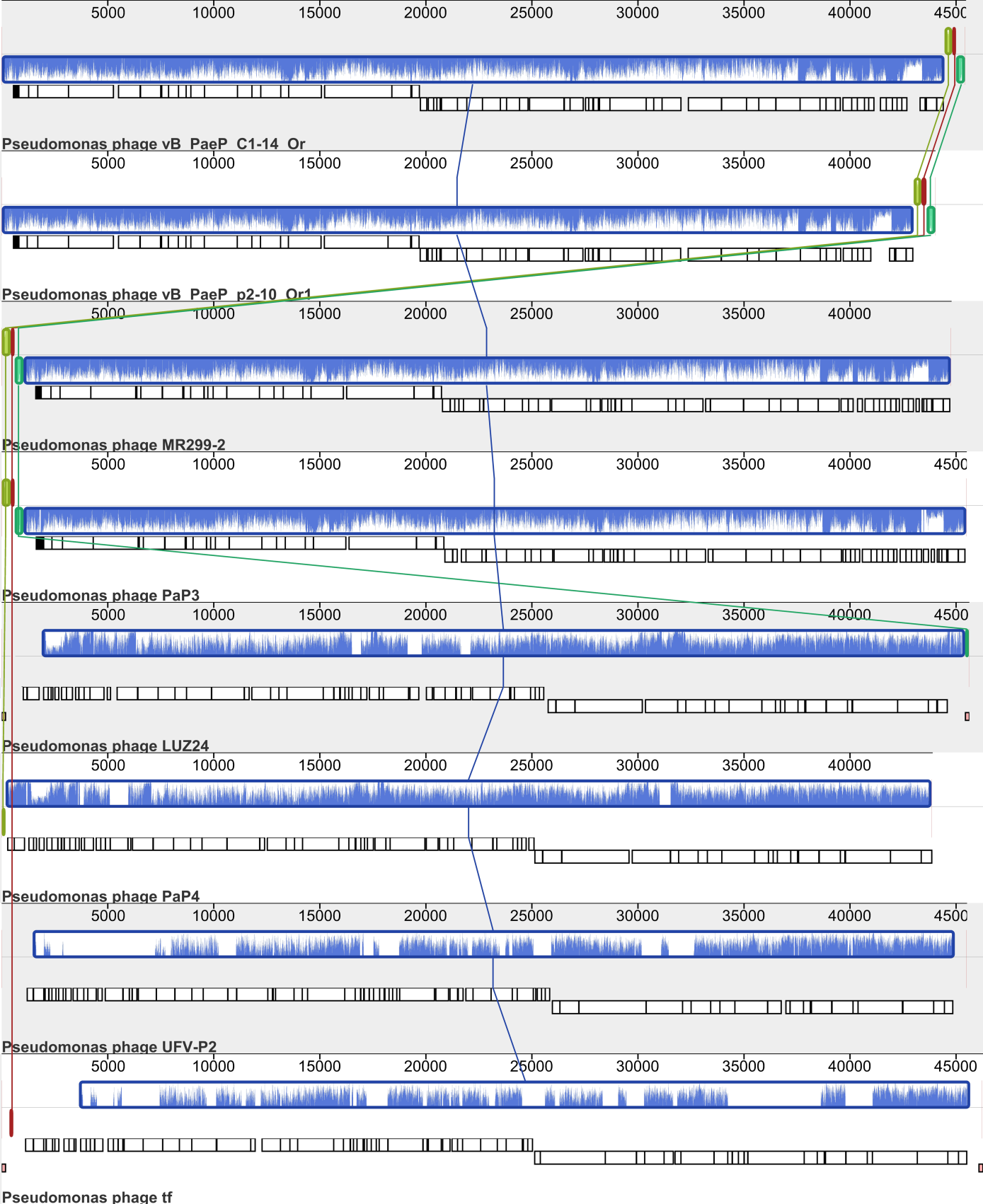


**Figure S2.** Comparison of the genomes of phages classified in LUZ24likevirus genus. The collinearity among genomes is represented by the conserved locally collinear blocks (LCBs). In the main block (blue), the regions of similarity plot with high identity corresponds to the set of shared LCBs (see Figure 2). The connection line between blocks correspond to the central point of LCB of the reference genome (phage LUZ24 genome).
